# Ubiquitination of Nonhistone Proteins in Cancer Development and Treatment

**DOI:** 10.3389/fonc.2020.621294

**Published:** 2021-02-11

**Authors:** Xiuzhen Zhang, Tong Meng, Shuaishuai Cui, Ling Feng, Dongwu Liu, Qiuxiang Pang, Ping Wang

**Affiliations:** ^1^ School of Life Sciences, Shandong University of Technology, Zibo, China; ^2^ Tongji University Cancer Center, Shanghai Tenth People’s Hospital of Tongji University, School of Medicine, Tongji University, Shanghai, China; ^3^ School of Agricultural Engineering and Food Science, Shandong University of Technology, Zibo, China

**Keywords:** ubiquitination, E3 ligase, deubiquitinase, nonhistone protein, cancer, proteolysis-targeting chimeras

## Abstract

Ubiquitination, a crucial post-translation modification, regulates the localization and stability of the substrate proteins including nonhistone proteins. The ubiquitin-proteasome system (UPS) on nonhistone proteins plays a critical role in many cellular processes such as DNA repair, transcription, signal transduction, and apoptosis. Its dysregulation induces various diseases including cancer, and the identification of this process may provide potential therapeutic targets for cancer treatment. In this review, we summarize the regulatory roles of key UPS members on major nonhistone substrates in cancer-related processes, such as cell cycle, cell proliferation, apoptosis, DNA damage repair, inflammation, and T cell dysfunction in cancer. In addition, we also highlight novel therapeutic interventions targeting the UPS members (E1s, E2s, E3s, proteasomes, and deubiquitinating enzymes). Furthermore, we discuss the application of proteolysis-targeting chimeras (PROTACs) technology as a novel anticancer therapeutic strategy in modulating protein target levels with the aid of UPS.

## Introduction

Post-translational modification with ubiquitin plays an important role in the regulation of protein degradation and turnover. Ubiquitin, a small protein of 76 amino acids, can be covalently attached to target proteins to form mono- or polyubiquitinated types. This process occurs by a cascade of enzymatic reactions including E1-activating enzymes, E2-conjugating enzymes, and E3 ubiquitin ligases. Polyubinquitin with different chain topologies on specific lysine residues on substrates is related to different functional consequences (1). Generally, polyubiquitin chains linked at the 48 lysine site (K48) or K11 site lead to 26S proteasome-mediated proteolysis, which plays an essential role in maintaining protein homeostasis, regulating cell cycle, and apoptosis. On the other hand, chains with K63 site, as well as monoubiquitination, representing non-proteolytic ubiquitination, participate in diverse cellular processes, such as signal transduction, autophagy, and DNA damage repair (2, 3). As for most substrates, they are first covalently modified by ubiquitin and then directed to the proteasome to be degraded. Also, the function of ubiquitin ligases can be reversed by deubiquitinating enzymes (DUBs), which remove ubiquitin from substrate proteins and participate in the regulation of various cellular pathways (4).

Ubiquitination is ubiquitous, and second only to phosphorylation in abundance (5). Some reports have shown that histone ubiquitination regulating DNA-driven processes such as gene transcription and DNA damage repair (6, 7), and aberrant histone ubiquitination frequently occurs in cancers (8). Accumulating evidences indicate that ubiquitylation of nonhistone proteins plays an important role in many cellular processes, including DNA repair, transcription, signal transduction, autophagy, apoptosis, and so on (9). Nonhistone protein substrates for ubiquitination include general transcription factors, transcriptional activators or repressors, nonhistone chromatin-associated protein, and nuclear receptor coactivators. Dysregulation of nonhistone lysine ubiquitination is closely associated with various human cancers (10). Therefore, it is more important to study the role of nonhistone ubiquitination in tumorigenesis and tumor treatment. Moreover, interrogating the regulatory networks of UPS can offer a strategy for delineating the mechanism of cancer development and facilitate the identification of therapeutic targets. Meanwhile, the UPS exhibits high substrate specificity, which makes targeting it a promising strategy for cancer treatment. Nowadays, many UPS inhibitors such as bortezomib, carfilzomib and ixazomib, have been well applied in cancer treatment (11, 12). In this review, we summarize the regulatory roles of key UPS members on major nonhistone substrates in cancer-related processes.

Recently, a novel strategy named proteolysis-targeting chimeras (PROTACs) has been developed. PROTAC is a strategy that utilizes a hybrid molecule (a short peptide or a small molecule) to link a specific protein to an E3 ubiquitin ligase and induces the targeted protein degradation by the UPS in the cell (13). PROTACs link the target protein to an E3 ubiquitin ligase by a designed hybrid molecule, providing a path for ubiquitinating undruggable proteins such as transcription factors, scaffolding proteins and nonenzymatic proteins. Due to their high selectivities, low working concentrations, and less off-target toxicities, PROTACs may boost the development of drug discovery (14).

Considering the importance of UPS in the regulation of cancer development and treatment, we focus on the regulatory roles of key UPS members on nonhistone proteins in cancer development and highlight the novel therapeutic options targeting them. In addition, we also discuss and summarize the applications and recent advances of PROTAC technology focusing on nonhistone proteins.

## The Ubiquitination Cascade and Deubiquitination

### The enzymes of Ubiquitination and Deubiquitination

The UPS contains a series of essential components: ubiquitin, E1s, E2s, E3s, DUBs, and the 26S proteasome. Until now, two E1s and about 40 E2s have been discovered, with more than 600 E3s conferring the diversity of protein substrates (15). Generally, E3 ligases are structurally classified into three subtypes: really interesting new gene (RING), homologous to E6-associated protein C-terminus (HECT) and RING-in-between-RING (RBR) E3s. RING E3 ligases are most abundant with more than 600 members in humans. About 30 HECT E3 ligases have been found in humans, including the NEDD4 family, the HERC family and other HECTs. RBR E3s have 14 members and work as hybrids of RING E3s and HECT E3s (16). In addition, there are approximate 100 DUBs and they are subdivided into 6 families based on sequence and structural similarity namely ubiquitin-specific proteases (USPs), ubiquitin carboxy-terminal hydrolases (UCHs), ovarian-tumor proteases (OTUs), Machado-Joseph disease protein proteases (MJD), JAB1/MPN/MOV34 metalloenzymes (JAMMs), and monocyte chemotactic protein-induced proteases (MCPIPs) (17). To date, more than 40 DUBs have been implicated in tumorigenesis (4).

### The Process of Ubiquitination and Deubiquitination

**Figure 1:**
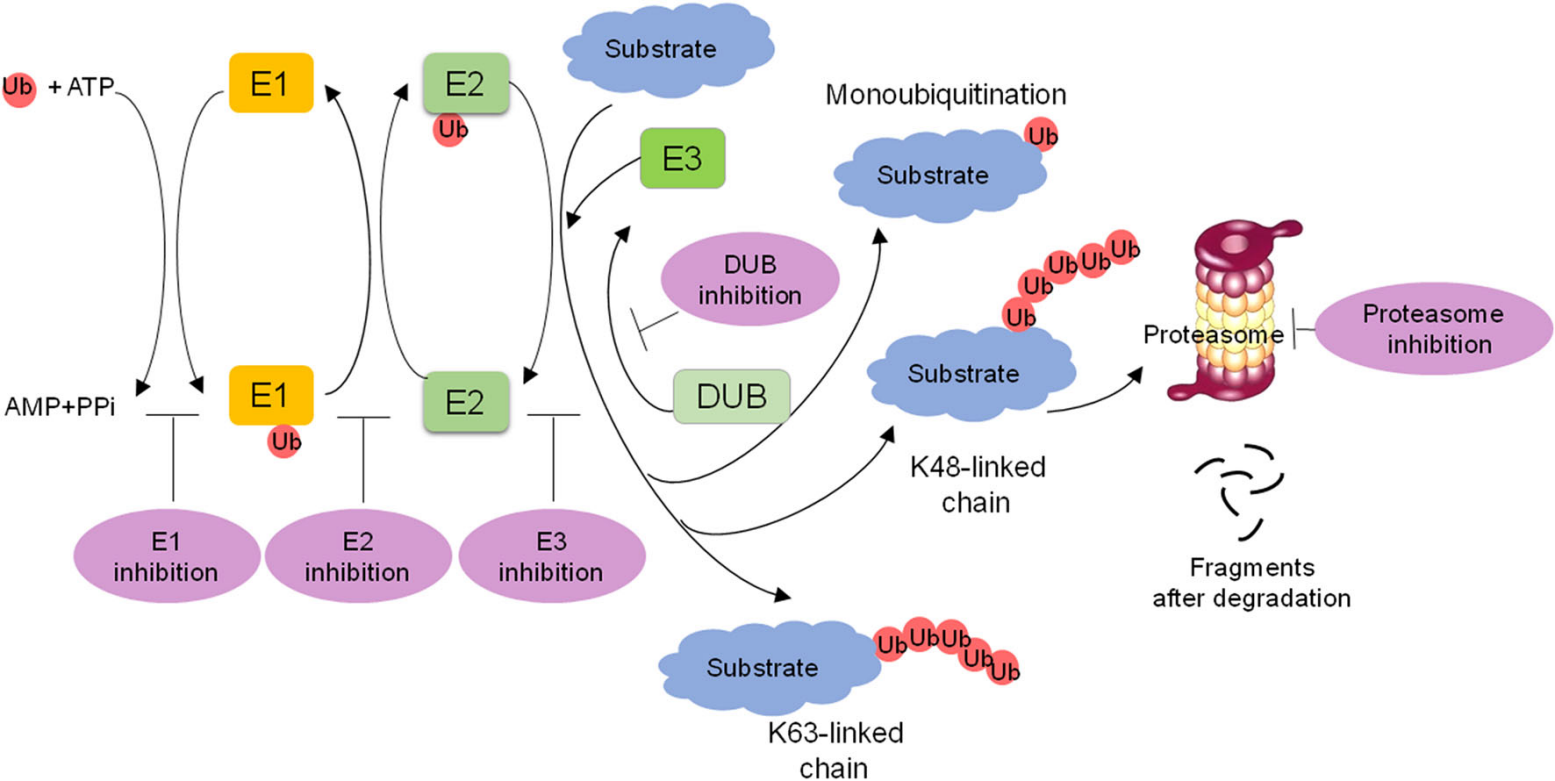
Overview of the ubiquitin-proteasome system (UPS) and targeting strategies for the UPS. The ubiquitin is activated with E1 in an ATP dependent manner, transferred to E2, and then transferred to the substrate through E3 ligase recognization, forming a mono- or polyubiquitinated protein. K48 or K11 polyubiquitin chains lead to 26S proteasome-mediated degradation. Monoubiquitination or K63 polyubiquitin chains are nonproteolytic ubiquitination signals and participate in many biological processes. DUBs remove or edit ubiquitins from substrate proteins. The targeting of E1s, E2s, E3s, proteasome and DUBs is a promising strategy for cancer treatment.

The process of ubiquitylation contains three steps (**Figure 1**). Initially, the α-carboxyl group of the C-terminal glycine residue of ubiquitin links to a cysteine residue on E1 in an ATP-dependent manner, and a thioester bond is formed. Subsequently, E2 binds to the activated ubiquitin, and the complex of E1 and ubiquitin is transferred to the catalytic cysteine of E2 *via* a trans(thio)esterification reaction. Finally, E3 recognizes the substrate and catalyzes the linking of ubiquitin to a specific lysine residue on the substrate. The function of E3 ligases can be reversed by DUBs, which mediate the removal and processing of ubiquitin. DUBs regulate multiple biological processes including the cell cycle, DNA repair, apoptosis, inflammation, and signaling pathways.

## The Roles of E3 ligases and DUBs in Regulating Cancer Development

The UPS regulates diverse important cellular processes including cell cycle arrest, cell proliferation, and apoptosis. Thus, dysregulation of its key members and their regulatory network is often associated with human diseases, particularly cancer. Increasing studies have revealed that E3 ligases and DUBs are involved in cancer development through various biological processes, such as cell cycle, cell proliferation, apoptosis, DNA damage repair, inflammation, and T cell dysfunction in cancer and some of them are shown in **Tables 1** and **2** (15).

**Table 1:** Some E3s involved in cancers.

E3	Substrate	Category	Associated cancer or cancer line	Biological functions	Model	Alteration in tumors	Reference
APC/C- CDC20	Cyclin A, cycin B1, securin,	Oncogene	Colorectal cancer	Cell cycle regulation	*In vivo*	Overexpression	(18)
APC/C- CDH1	CDC20, CDC25A	Tumor suppressor	Breast cancer	Cell cycle regulation	*In vivo*		(19)
SCF^FBXW7^	c-Myc, c-Jun, cyclin E, mTOR, Notch-1, Mcl-1,	Tumor suppressor	Metastatic colorectal denocarcinoma, T-cell acute lymphoblastic leukemia, and cholangiocarcinomas	Cell cycle regulation,	*In vivo*	Mutation	(20–23)
	c-Myc	Tumor suppressor	Leukemia-initiating cell	Cell proliferation	*In vitro*	Mutation	(24, 25)
SCF^SKP2^	p27, p21, p57, cyclin A, cyclin E, cyclin D1	Oncogene	Breast cancer lung cancer	Cell cycle regulation	*In vivo*	Overexpression	(26)
	c-Myc,			Cell proliferation	*In vivo*	Overexpression	(27)
SCF^βTrCPs^	Mcl-1, BimEL, PDCD4, STAT1	depends on substrates	Colorectal cancer, pancreatic cancer	Cell cycle regulation	*In vivo*	Overexpression	(28, 29)
Parkin	cyclin D, cyclin E	Tumor suppressor	Glioma, colorectal cancer	Cell cycle regulation	*In vivo*	Mutation	(30, 31)
MDM2	Retinoblastoma protein, p53	Oncogene	Lung cancer, colorectal cancer, cutaneous melanoma, breast cancer	Cell cycle control, Apoptosis	*In vivo*	Overexpression, Mutation	(32, 33) (34),
TRPC4AP/ TRUSS	c-Myc		IMR5 neuroblastoma cells, U2OS, HeLa cells	Cell proliferation	*In vitro*		(35)
KCTD2	c-Myc		Glioma stem cells	Cell proliferation	*In vitro*	Suppression	(36)
CHIP	c-Myc		Glioma	Cell proliferation			(37)
HectH9	c-Myc	Oncogene	HeLa, T47D, MCF7, MRC5 cells	Cell proliferation	*In vivo*, *In vitro*	Overexpression	(38)
hUTP14a	c-Myc	Oncogene	Colorectal cancer	Cell proliferation	*In vivo*	Upregulation	(39)
	p53, retinoblastoma protein	Oncogene	U2OS cell, H1299, HCT116 cell	Apoptosis	*In vitro*, *In vivo*	Upregulation	(40, 41)
TRAF6	TAB2			Inflammation			(42)
Fbxo38	PD-1	Tumor suppressor		T cell dysfunction in cancer	*In vivo*	Downregulation	(43)
Stub1,Cbl-b	Foxp3	Tumor suppressor	Colitis	Inflammation	*In vivo*	Downregulation	(44, 45)
VHL	HIF-1α	Tumor suppressor	Pancreatic cancer	Inflammation			(46)

**Table 2:** Some DUBs involved in cancers.

DUB	Substrate	Category	Associated cancer or cancer line	Biological functions	Model	Alteration in tumors	Reference
BAP1	HCF-1	Tumor suppressor	OCM1 cell, NCI-H226 lung carcinoma cell line	Cell proliferation	*In vitro*	Loss, mutation	(47–49)
OTUD7B/ Cezanne	APC/C	Oncogene	HCT116, RPE1, HeLaS3, U2OS cells	Cell proliferation	*In vitro*	Overexpression	(50)
USP21	FOXM1, p53	Oncogene	Breast cancer	Cell cycle progression, NF-κB signing	*In vitro*, *In vivo*	Overexpression	(51)
	BRCA2	Oncogene	Hepatocellular carcinoma	DNA damage repair, NF-κB signaling	*In vivo*	Overexpression	(52)
USP5	FoxM1	Oncogene	Pancreatic cancer	Cell cycle regulation		Overexpression	(53, 54)
USP2	Cyclin D1, MDM2	Oncogene	Hepatoma and breast cancer cells	Cell cycle regulation, apoptosis	*In vitro*	Overexpression	(55, 56)
USP14	Cyclin B1		Breast cancer, colorectal cancer, non-small cell lung cancer	Cell cycle regulation	*In vitro*		(57)
USP44	Cdc20	Oncogene	HeLa cell, T-cell leukemias	Cell cycle regulation	*In vitro*, *In vivo*	Overexpression	(58, 59)
USP37	Cyclin A,		U2OS cells, HeLa cells, lung cancer	Cell cycle regulation, apoptosis	*In vitro*, *In vivo*	Overexpression	(60, 61)
USP7	Retinoblastoma protein, p53, MDM2, FOXO4	Tumor suppressor	HEK293, prostate cancer, colon cancer, non-small cell lung cancer	Cell cycle arrest, apoptosis, Cell proliferation	*In vitro*, *In vivo*	Downregulation	(62, 63) (32, 34),
USP11	BRCA2	Oncogene	U2OS cell, breast cancer	DNA damage repair,	*In vitro*, *In vivo*	Upregulation	(64, 65)
USP13	RAP80	Oncogene	Ovarian cancer	DNA damage response	*In vivo*	Overexpression	(66)
USP22	c-Myc	Tumor promoter	Breast cancer	apoptosis	*In vivo*	Overexpression	(67)
USP28	c-Myc	Oncogene	Colon cancer, breast cancer	Cell cycle regulation, apoptosis, DNA damage repair	*In vivo*	Overexpression	(68)
USP36	c-Myc	Oncogene	Breast cancer, lung cancer	Apoptosis	*In vivo*	Overexpression	(69)
USP10	p53	Tumor suppressor	HCT116 cell	DNA damage repair	*In vitro*	Downregulation	(70)
A20	TRAF2, TRAF6, RIP1	Tumor suppressor	B-cell lymphomas	inflammation, apoptosis inflammation	*In vivo*	Downregulation	(71)
CYLD	IκK	Tumor suppressor	Cylindromatosis	inflammation	*In vivo*	Downregulation	(72)

### E3 ligases and DUBs Regulate Cell Cycle

**Figure 2:**
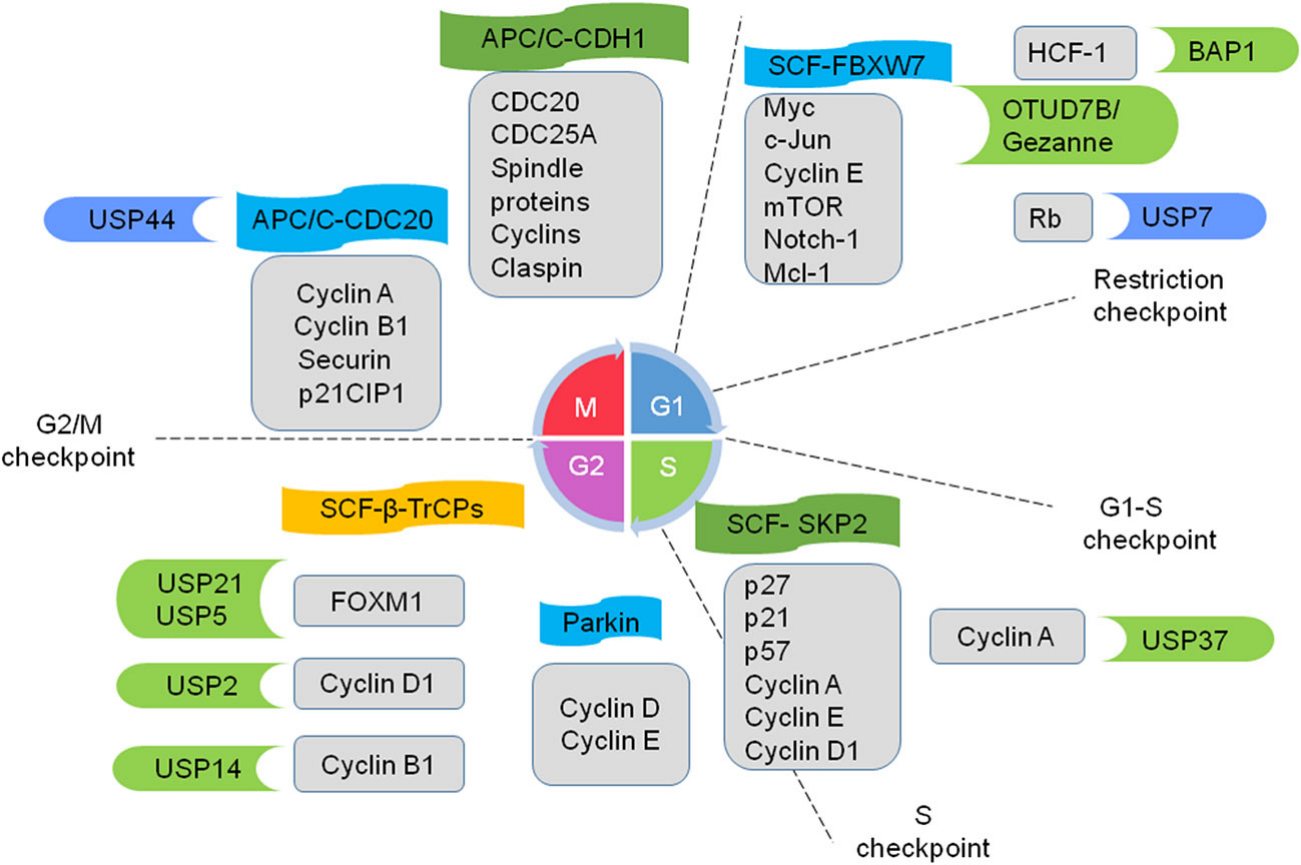
Ubiquitin ligases and DUBs coordinate to regulate cell cycle progression. E3 ligase APC/C (anaphase-promoting complex; also named as the cyclosome) recruits cell division cycle 20 (cdc20) and CDC20-like protein 1 (CDH1). APC/C-CDC20 promotes cell cycle transition from metaphase to anaphase, while APC/C-CDH1 mediates mitotic exit and early G1 entry. E3 ligases SCF (S-phase kinase-associated protein 1-cullin 1-F-box protein) complexes work on a subset of cyclins and CDK inhibitors and regulate progression from G1 to the onset of mitosis. FBXW7, SKP2, and β-TrCPs are well-studied F-box proteins. E3 Parkin downregulates some G1/S kinases. Several DUBs play crucial roles in cell-cycle progression in cancers. Some example substrates of E3 and DUBs are shown in the gray boxes. The E3 and DUBs in green are tumor promoters and the ones in blue are tumor suppressors.

Cell cycle progression and arrest are commonly deregulated in cancer (73). Increasing evidence indicates that multiple E3s participate in regulating cell cycle progression (**Figure 2**). Thus, the deregulation of E3s leads to the sustained proliferation and genomic instability of cancer cells. The anaphase-promoting complex named the cyclosome (APC/C) is the most sophisticated RING E3 ligase. It precisely governs cell cycle progression by recruiting cell division cycle 20 (CDC20) and CDC20-like protein 1 (CDH1) in turns. APC/C-CDC20 regulates cell cycle transition from metaphase to anaphase, while APC/C-CDH1 mediates mitotic exit and early G1 entry. Many studies indicate that Cdh1 functions as a tumor suppressor, whereas CDC20 may function as an oncoprotein to promote the development and progression of cancers (18, 74).

Another representative example is SCF E3 ligases, which consist of four components: S-phase kinase-associated protein 1 (SKP1), cullin 1, Roc1/Rbx1/Hrt1 and an F-box protein (FBP). Commonly, FBPs serve for substrate recognition in the complexes and selectively regulate diverse biological processes (19). FBXW7, F-box/WD repeat-containing protein 7 (FBXW7), S-phase kinase associated protein2 (SKP2), and β-transducin repeat containing proteins (β-TrCPs) are well-studied FBPs. FBXW7 a tumor suppressor, works on many oncogenes including Myc, c-Jun, cyclin E, mTOR, Notch-1 and Mcl-1. It is often mutated or deleted in lots of cancers such as metastatic colorectal adenocarcinoma, T-cell acute lymphoblastic leukemia, and cholangiocarcinomas (20–22, 75). SKP2 plays a critical role during S and G2/M phases through regulating some cell cycle proteins, such as p21, p57, cyclin A, cyclin E, cyclin D1, and CDK inhibitors (e.g. p27). SKP2 is an important oncogene and is widely overexpressed in various cancers, such as breast cancer (23) and hepatocellular carcinoma (26). β-TrCPs-containing SCF complexes play a dual role in cell cycle checkpoint control: mediating and relieving cell cycle arrest *via* bonding different substrates (28, 76). Thus, the SCF complexes work on a subset of cyclins and CDK inhibitors to regulate the progression from G1 to the onset of mitosis. In addition, Parkin, a well-known RBR E3 ligase, controls the cell cycle by downregulating some G1/S kinases such as cyclin D and cyclin E (29, 30).

DUBs also participate in the regulation of cell-cycle progression (**Figure 2**) (31). For instance, E2F transcription factors play a key role in cell-cycle progression through G1 and into S-phase (77). The tumor suppressor retinoblastoma protein (Rb) maintains the cell in G1 through inhibiting E2F (78). However, hyperphosphorylated Rb dissociates from E2F, leading to the transcription of S-phase genes. The E3 ligase MDM2 promotes Rb degradation *via* ubiquitylation (79). On the contrary, the DUB USP7 directly reverses MDM2-mediated polyubiquitylation of Rb, stalling the cell cycle in G1 and inhibiting cell proliferation (32). Tumor suppressor BRCA1-associated protein 1 (BAP1), whose mutations can be seen in many cancers (62), has been found that it also could promote cell proliferation through deubiquitylating host cell factor 1 (HCF-1). HCF-1, an important transcriptional co-regulator of E2F, promotes cell cycle progression at the G1/S boundary by activating the E2F1 transcription factor. Therefore, BAP1 regulates cell proliferation at G1/S by co-regulating transcription from HCF-1/E2F-governed promoters. Moreover, BAP1 knockdown leads to G1 arrest and decreases the expression of S phase genes in OCM1 cells and NCI-H226 lung carcinoma cell line (47, 48, 80). It is well known that APC/C plays a crucial role in the completion of mitosis and maintenance of G1. Recently, OTUD7B/Cezanne has been reported to deubiquitinate and stabilize the APC/C substrates, as well as promote mitotic progression and cell proliferation. Cezanne is upregulated in multiple tumors, suggesting a potential role in cancer cell proliferation (49). Besides, the transcription factor FOXM1 participates in cell cycle progression and is upregulated in basal-like breast cancer. Arceci et al. reveal that USP21 directly binds to FOXM1, makes it deubiquitinate, and increases its expression level *in vitro* and *in vivo*. Suppression of USP21 causes a mitotic entry delay to slow proliferation and sensitivity to paclitaxel in cell culture and animal xenografts (50). The deubiquitinating enzyme USP5 is overexpressed in numerous malignancies, promoting tumor growth *via* modulating cell cycle regulators such as FoxM1. USP5 deficiency also induces DNA damage, cell cycle arrest and apoptosis in pancreatic ductal adenocarcinoma cells (51, 53). Besides, USP2 and USP14 regulate the cancer cell cycle *via* deubiquitinating cyclin D1 (54) and Cyclin B1 (55), respectively. Knocking down USP14 arrests cell cycle at the G2/M phase and inhibits the proliferation and migration of breast cancer cells (55), USP44 deubiquitinates the APC-inhibitory Mad2-Cdc20 complex, thereby preventing anaphase onset (57, 58). USP37 deubiquitinates and stabilizes Cyclin A and promotes S phase entry (59).

### E3 Ligases and DUBs Regulate Cell Proliferation

Many oncogenes can induce cancer cell proliferation, and UPS mediates their transcription by modulating general transcription factors, transcriptional activators and transcriptional coactivators *via* proteolytic and nonproteolytic ubiquitination (60). Here, we take the oncogene c-Myc as an example to show how ubiquitination regulates the transcription of oncogenes in cancer.

The overexpression of c-Myc is widely found in many cancers and is related to cell growth, proliferation, apoptosis and metabolic pathways (81). Its accumulation is also associated with poor cancer outcomes (82). Myc levels are controlled through targeted degradation by UPS (83). Multiple E3s are involved in modulating c-Myc activity in a tissue-specific manner. For instance, the ubiquitin ligase SCF-FBXW7 directly catalyzes c-Myc ubiquitination in a glycogen synthase kinase 3 phosphorylation-3-dependent manner and leads to c-Myc degradation *in vitro* (84). Furthermore, FBXW7 regulates the ubiquitylation of c-Myc protein and mediates leukemia-initiating cell activity (24). TRPC4AP (transient receptor potential cation channel, subfamily C, member 4-associated protein)/TRUSS (tumor necrosis factor receptor-associated ubiquitous scaffolding and signaling protein) binds to c-Myc and promotes its ubiquitination and degradation in multiple cancer cells (25). CRL3-potassium channel tetramerization domain-containing 2 (KCTD2) mediates c-Myc protein degradation by ubiquitination and suppresses gliomagenesis (35). E3 ligase CHIP interacts and degrades c-Myc by ubiquitination in glioma cells (36). In addition, 11S proteasomal activator REGγ has been reported to induce the degradation of c-Myc in cancer cells (37). On the other hand, SCF-SKP2 enhances c-Myc transcriptional activity by enabling the formation of c-Myc activator complexes (85). The E3 ligase HectH9 regulates the transcriptional activation of Myc through forming a lysine 63-linked polyubiquitin chain and promotes tumor cell proliferation *in vivo* and *in vitro* (27).

The deubiquitinating enzymes can prevent c-Myc degradation, maintain its stability, and then promote cancer progression. USP28 was the first DUB shown to regulate c-Myc stability. It is highly expressed in colon and breast carcinomas and binds to Myc through interacting with FBW7alpha to stabilize Myc in the nucleus (38). USP22 increased c-Myc stability *via* deubiquitination in breast cancer cells (68). We previously found that USP37 was significantly upregulated in human lung cancer tissues, and directly deubiquitinated and stabilized c-Myc independent of Fbw7 (67). USP36, a highly expressed USP in a subset of human breast and lung cancers, could interact with the nucleolar Fbw7γ and maintain c-Myc stability in the nucleolus (61). Recently, a novel E3 ligase, human U three protein 14a (hUTP14a) is upregulated in human colorectal cancer tissues, and it stabilizes c-Myc through forming a complex with USP36/Fbw7γ in the nucleolus and promote cancer progression (69).

### E3 Ligases and DUBs Regulate Apoptosis

Apoptosis could inhibit aberrant cell cycle progression and prevent tumorigenesis (39). If apoptotic pathways are abrogated, the cells may not appropriately induce apoptosis, which may lead to tumorigenesis. As a tumor suppressor protein, p53 is frequently mutated in most cancers and plays a pivotal role in apoptosis, genome instability and mutation. Ubiquitination has been found to play a key role in regulating p53 degradation as well as its activity and localization. For instance, MDM2 (murine double minute 2) has been found to negatively regulate p53 with diverse mechanisms. It can interact directly and degrade p53 *via* ubiquitination. Besides, it can connect p53 and pRb to form an Rb-Mdm2-p53 trimeric complex for the regulation of p53-induced apoptosis (86). Mdm2 can also form a heterodimer with MdmX (Mdm4) and participate in ubiquitin-mediated p53 degradation (33). Moreover, Mdm2 is upregulated in multiple cancers such as colorectal cancer, cutaneous melanoma and breast cancer (63). Therefore, the inhibition of p53-MDM2 interaction facilitates p53-mediated cell-cycle arrest or apoptosis in cancer cells.

Up to now, many DUBs are involved in the regulation of p53. For example, USP7 modulates the stability of both p53 and MDM2, and maintains the level of p53 ubiquitylation (34, 87); USP2 affects the stability of MDM2 (88); Otub1 inhibits p53 ubiquitination and activates p53 in cells (56); USP10 regulates the location and stability of p53, and stabilize both mutated and wild-type p53, thereby having a dual role in tumorigenesis (89).

Several E3s target anti-apoptotic protein myeloid cell leukemia 1 (MCL1) and sensitize cells to apoptosis. For example, DNA damage promotes HUWE1 bind to MCL1 and marks MCL1 for proteasomal degradation; the cell cycle regulators APC/C-CDC20 and SCF-FBXW7 degrade MCL1 and link apoptosis to prolonged mitotic arrest. Human UTP14a is upregulated in several types of tumors and involved in tumor progression *via* multiple mechanisms. It also exhibits an anti-apoptotic activity through the intrinsic apoptotic pathway, and protects tumor cells from chemotherapeutic drug-induced apoptosis (70). It binds p53 and induces p53 degradation through a ubiquitin-independent manner (40). Moreover, hUTP14a can also bind tumor suppressor pRb, and promote the polyubiquitination and degradation of pRb *in vitro* and *in vivo* (90). Thus hUTP14a might possess the potential as a target for anti-tumor therapy.

### E3 ligases and DUBs Regulate DNA Damage Repair

Errors in DNA replication and repair often cause genomic instability (73). DNA damage repair is critical to maintain genome integrity and prevent cancer. Many E3s including MDM2 and BRCA1 participate in regulating the DNA damage response and cell cycle checkpoints to cancer development. In brief, DNA double-strand breaks (DSBs) induce the activation of DNA damage sensors, which leads to the inactivation of MDM2, maintenance of p53 stability, promotion of SCF-β-TrCP mediated degradation of CDK phosphatase, and decrease of CDK activity. In the meantime, DNA repair machines are recruited to DNA damage sites under the control of ubiquitination. The inhibition of homologous recombination (HR) during G1 is also dependent on ubiquitylation mediated by APC/C-CDH1 and cullin 3-RING-E3 ligase (CRL3)-kelch-like ECH-associated protein 1 (KEAP1). USP11 is also involved in the regulation of DNA double-strand break repair, which is often up-regulated in cancer, resulting in resistance to poly ADP ribose polymerase 1 (PARP) inhibitors (41, 64). USP21 deubiquitinates and stabilizes BRCA2, promotes HR efficiency, and enhances homologous recombination efficiency and tumor cell growth (65). USP13 deubiquitinates receptor-associated protein 80 (RAP80) and promotes DNA damage response. Therefore, inhibiting USP13 makes ovarian cancer cells sensitive to cisplatin and olaparib (a PARP inhibitor) (52).

### E3 ligases and DUBs Regulate Inflammation

**Figure 3:**
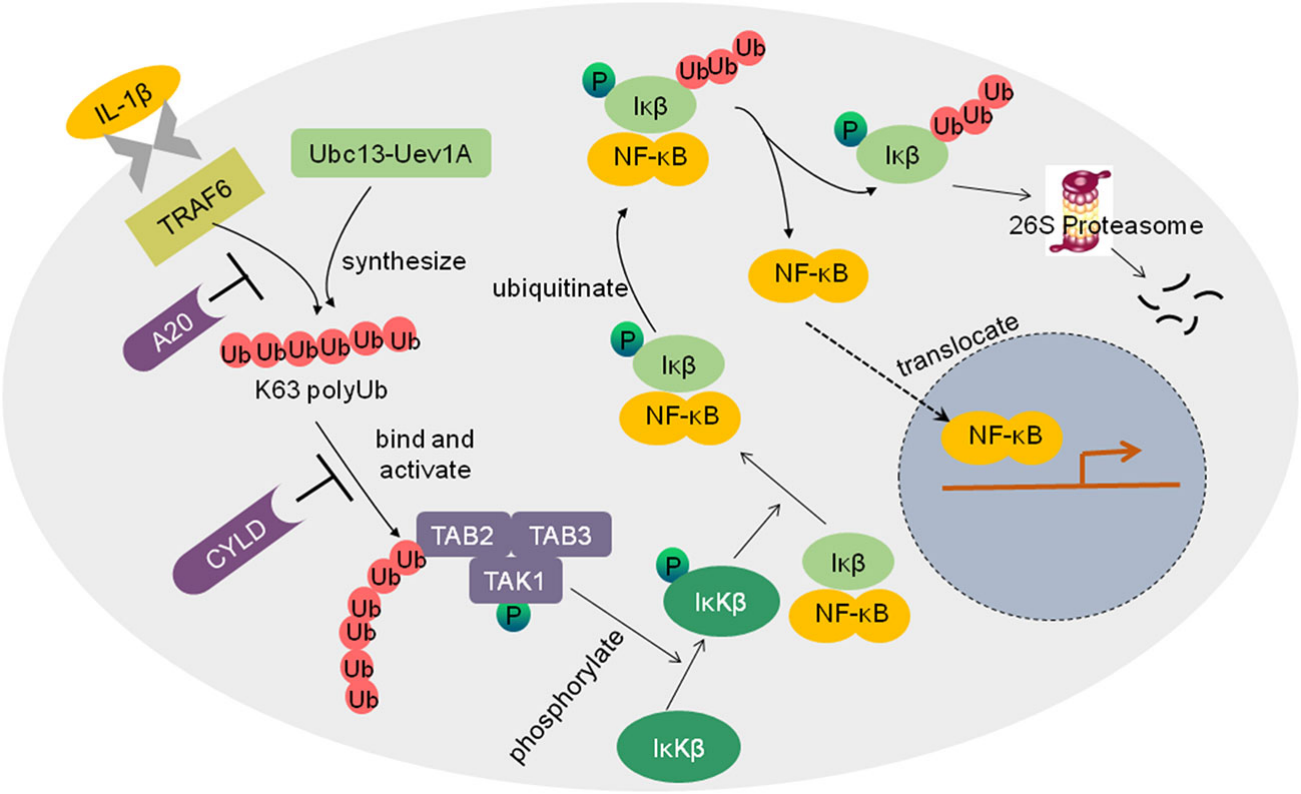
Schematic diagram of the regulation of NF-kB activation by ubiquitin ligases and DUBs. IL-1β activates the ubiquitin E3 ligase TRAF6, TRAF6 cooperated with the E2 enzyme Ubc13-Uev1A to synthesize K63 polyubiquitin chains and add them to the TAB2 subunit of the TGF-β activated kinase 1 (TAK1) kinase complex, which results in TAK1 activation. TAK1 then phosphorylates IκKβ. Phosphorylated IκB is subsequently ubiquitinated and degraded by 26S proteasome, thus allowing NF-κB to translocate to the nucleus, and the NF-κB pathway is activated. Deubiquitinases such as A20 and CYLD inhibit the activation of the NF-κB pathway.

Cancer-related inflammation plays an important role in tumor development and progression. The transcription factor NF−κB regulates multiple biological processes including inflammation, immunity, cell proliferation and apoptosis. Abnormal activation of NF-κB has been involved in tumorigenesis. Ubiquitination regulates NF-κB pathways in proteasome-dependent and independent mechanisms (**Figure 3**) (66). For example, NF-κB is activated by the inflammatory cytokine interleukin-1 (IL-1). Without simulation, NF-κB is inactive in the cytoplasm binding to the inhibitory proteins of the κB family (IκB). IL-1β activates the ubiquitin E3 ligase tumor necrosis factor receptor-associated factor 6 (TRAF6). TRAF6 cooperates with the E2 enzyme Ubc13-Uev1A to synthesize K63 polyubiquitin chains and adds them to the TAB2 (TGFβ-activated kinase 1 binding protein 2) subunit of the TGF-β activated kinase 1 (TAK1) kinase complex, resulting in TAK1 activation. TAK1 then phosphorylates the IκB kinase β (IκKβ). Phosphorylated IκB is subsequently ubiquitinated and degraded by 26S proteasome, thereby allowing NF-κB to translocate to the nucleus and activate gene expression.

Inappropriate activation of NF-κB has been linked to cancers. NF-κB activation could be tightly controlled by deubiquitinating enzymes as negative regulators of IκK. For example, DUB A20 inhibits IκK activation *via* three mechanisms, replacing K63 polyubiquitin chains from receptor-interacting protein 1(RIP1) with K48 polyubiquitin chains, blocking the interaction between Ubc13 and TRAFs, and inhibiting IκK phosphorylation by TAK1 (42). Another well-known DUB is the tumor suppressor CYLD, which inhibits NF-κB activation by cleaving K63 as well as linear polyubiquitin chains to inhibit IκK (71). Lack of CYLD in cells would elevate NF-κB activation, which likely contributes to tumor development.

### E3 ligases and DUBs Regulate T Cell Dysfunction in Cancer

T cell activation is critical for the initiation and regulation of the immune response in cancer immunotherapy. It requires at least two signals to become fully activated. One occurs after the engagement of the T cell receptor (TCR) and major histocompatibility complex (MHC). Another is provided when co-stimulator CD28 binds to CD80 and CD86 that are expressed on antigen-presenting cells (APCs). However, the multifaceted suppressive signals that existed in the tumor microenvironment make intratumoral T cells dysfunctional. The main traits of T cell dysfunction include some inhibitory receptors (e.g., PD-1), inhibitory cells (e.g., Treg cells), suppressive soluble mediators (e.g., TGFβ), transcriptional factors (e.g., T-bet), etc (72). UPS has been found to play a key regulatory role in maintaining T cell dysfunction with diverse mechanisms (91).

Dysfunctional T cells usually have abnormally high expression of multiple inhibitory receptors such as PD-1. Inhibitory receptors binding to their ligands negatively regulate an immune response. A recent study has identified that E3 ligase Fbxo38 ubiquitinates and degrades PD-1 in activated intratumoral T cells, which proves a novel mechanism for cancer immunotherapy. Fbxo38 can be activated by IL-2-induced STAT5 in activated T cells. In the dysfunctional T cells, Fbxo38 is downregulated, leading to an increased PD-1 abundance and impressive tumor immune response (92).

Regulatory T (Treg) cells are a subpopulation of CD4^+^ T cells that are crucial for maintaining immune tolerance. Treg cells usually produce immunosuppressive molecules such as TGFβ and inhibit the function of effector T cells. Treg cell development and function are determined by the transcription factor forkhead box protein 3 (Foxp3) and several E3s are involved in the process. For example, Stub1 and casitas B cell lymphoma protein b (Cbl-b) ubiquitinate Foxp3 and negatively regulate Treg cell development (43, 44). E3 ligase von Hippel-Lindau (VHL), Itchy homolog (Itch) and gene related to anergy in lymphocytes (Grail) participate in maintaining Treg cell repressive function (45, 46). Loss of VHL in Tregs leads to type 1 T helper (Th1)-like cell conversion and interferon-gamma (IFN-*γ*) production (45). Itch deficiency in Treg cells results in severe airway inflammation in mice, increasing TH2 cytokine production (46). Also, GRAIL-deficient Treg cells induce decreased suppressive function and increased Th17 cell-related gene expressions (93). Cbl-b and Grail have been found to play crucial roles in tumor immunosurveillance. Their loss inhibits tumor formation in mice. Cbl-b^-/-^ and Grail^-/-^ CD8^+^ T cells can be fully activated in the absence of costimulatory factors *in vitro*. They could promote tumor rejection and inhibit tumor formation when they are transferred into tumor-bearing mice (94, 95). These studies suggest that Cbl-b and Grail may serve as therapeutic targets to antitumor immunity.

TGFβ, a well-known immunosuppressor factor, plays an important role in immune tolerance (96). It not only promotes thymic Treg cell development by repressing T cell clonal deletion but also regulates peripheral Treg cell differentiation and maintains Treg cell function by inducing Foxp3 expression (96). Moreover, TGFβ inhibits T cell proliferation by decreasing IL-2 production and upregulating cell cycle inhibitors (97). It also blocks CD4^+^ T cell differentiation by modulating T-bet or GATA expression (97). Besides, TGFβ downregulates the expressions of cytolytic genes in cytotoxic T lymphocytes (98), costimulatory factors and MHC II molecules in dendritic cells and macrophages, reducing antigen resenting ability and regulating T cell function indirectly (99). In fact, as a versatile cytokine, TGFβ exerts pivotal functions in diverse processes of cancer development, such as proliferation, differentiation, apoptosis, and migration, depending on the target cells (100). Thus, TGFβ signaling has been regarded as a potential therapeutic target for the treatment of cancers.

**Figure 4:**
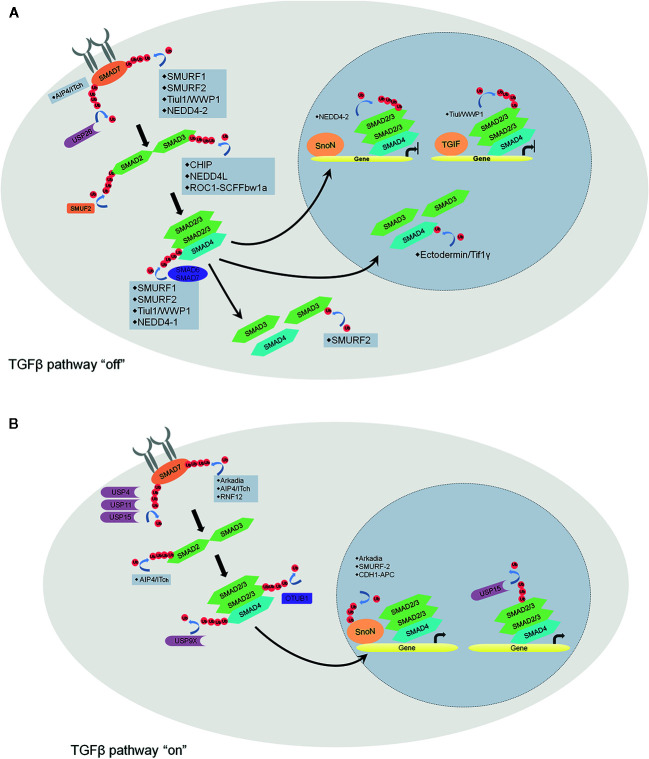
Schematic overview of the regulation of TGFβ pathway by ubiquitin ligases and DUBs. **(A)** factors that turn off the TGFβ pathway. AIP4/Itch brings SMAD7 to TGFβRI and prevents the activation of SMAD2. SMAD7 recruits E3 ligases SMURF1, SMURF2, Tuil1/WWP1, and NEDD4-2 to ubiquitinate and degrade the receptor complex. On the contrary, USP26 stabilizes SMAD7 *via* deubiquitination. As for SMADs, SMURF2, and NEDD4-2 target SMAD2 for degradation whereas SMAD3 is targeted by E3 ligases CHIP and ROC1-SCFFbw1a. SMAD4 is regulated by E3 ligases SMURF1, SMURF2, Tuil1/WWP1, and NEDD4-2 through forming a complex with SMAD7, SMAD6 or activated SMAD2. The R-SMAD/SMAD4 complex can be dissociated by SMURF2 monoubiquitinates SMAD3 or Ectodermin/Tif1γ monoubiquitinates SMAD4. Once the R-SMAD/SMAD4 complex enters the nucleus, the DNA-binding proteins SnoN and TGIF direct NEDD4-2 and Tiul/WWP1 to degrade SMAD2 and inhibit the signaling. **(B)** factors that turn on the TGFβ pathway. At the receptor level, USP4, USP11, and USP15 stabilize the receptor complex. E3s Arkadia, AIP4/Itch and RNF12 induce SMAD7 degradation. OTUB1 maintains the stability of SMAD2/3 and also promotes the R-SMAD/SMAD4 complex formation by preventing ubiquitination on R-SMAD. In the nucleus, transcriptional repressor SnoN can be degraded induced by E3s Arkadia, SMURF2 and CDH1-APC. Monoubiquitination of R-SMADs prevents the R-SMAD/SMAD4 complex binding with the DNA, while USP15 reverses the modification and promotes TGFβ-dependent transcription.

Dynamic ubiquitination/deubiquitination plays a key role in the regulation of the TGFβ signaling pathway (**Figure 4**) (101). The TGFβ1-induced TGFβ pathway activation consists of receptors (TGF receptor I and II), receptor-SMADs (SMAD2 and SMAD3), co-SMAD (SMAD4), and inhibitor adaptor SMAD (SMAD7). TGFβ1 binding induces TGFRII to phosphate TGFRI, and then the activated-TGFRI phosphorylates SMAD2 and SMAD3. Subsequently, the phosphorylated SMAD2/3 dissociates from the receptor and oligomerizes with SMAD4. Following that, SMAD2/3/4 translocates to the nucleus and recruits other gene regulatory proteins and transcript specific genes. Many E3s and DUBs are reported to be involved in turning off the TGFβ pathway. For example, AIP4/Itch brings SMAD7 to TGFβRI and prevents the activation of SMAD2 (102). SMAD7 also serves as a scaffold to recruit E3 ligases SMURF1, SMURF2, Tuil1/WWP1 and NEDD4-2 to ubiquitinate and degrade the receptor complex (103–106). On the contrary, USP26 stabilizes SMAD7 *via* deubiquitination (107). As for SMADs, SMURF2 and NEDD4-2 target SMAD2 for degradation (106, 108) whereas SMAD3 is targeted by E3 ligases CHIP and ROC1-SCFFbw1a (109, 110). SMAD4 is indirectly regulated by E3 ligases SMURF1, SMURF2, Tuil1/WWP1, and NEDD4-2 through forming a complex with SMAD7, SMAD6 or activated SMAD2 (111). SMAD4 has a point mutation in many cancers. In this case, these protein variants are degraded by E3 ligases SCF-Skp2 and SCF-β-TrCP1 (112, 113) In addition, the R-SMAD/SMAD4 complex can be dissociated by SMURF2 monoubiquitinates SMAD3 or Ectodermin/Tif1γ monoubiquitinates SMAD4. Once the R-SMAD/SMAD4 complex enters the nucleus, the DNA-binding proteins SnoN and TGIF direct NEDD4-2 and Tiul/WWP1 to degrade SMAD2 and inhibit the signaling.

On the other hand, lots of E3s and DUBs participate in turning on the TGFβ pathway. At the receptor level, USP4 interacts directly with TGFβRI to maintain its stability (114). DUBs such as USP11 and USP15, stabilize the receptor complex by being associated with the scaffold protein SMAD7 (115, 116). SMAD7 can be degraded by E3 ligases Arkadia, AIP4/Itch and RNF12 mediated ubiquitination (117). OTUB1 maintains the stability of SMAD2/3 by reversing the ubiquitination of SMAD2 and USP9X, and also promotes the R-SMAD/SMAD4 complex formation by preventing ubiquitination on R-SMAD (118). In the nucleus, transcriptional repressor SnoN can be degraded by E3s Arkadia, SMURF2 and CDH1-APC mediated ubiquitination (119, 120). Monoubiquitination of R-SMADs prevents the R-SMAD/SMAD4 complex binding with the DNA, while USP15 reverses the modification and promotes TGFβ dependent transcription.

The T-box family transcription factor T-bet regulates the Th1 cell differentiation and induces the production of IFN-*γ*. Recently, it has been shown that it is expressed in Treg and participates in relevant immunosuppressive function (121). It has been suggested that T-bet is required in T cell dysfunction (72). Although the underlying mechanism of T-bet ubiquitination is unknown, USP10 has been found to stabilize T-bet *via* deubiquitination and enhance the secretion of IFN-*γ* (122).

Furthermore, UPS could regulate TCR activation. For instance, E3 ligases Cbl, Itch, and Grail degrade the TCR complex and inhibit T cell activation through proteolysis-dependent mechanisms (91, 123). In contrast, USP12 has been found to stabilize the TCR complex and promote TCR signaling through deubiquitylating TCR adaptor proteins LAT and Trat1 in primary mouse T lymphocytes (124). Naik et al. found that USP9X regulated TCR signaling and tolerance induction, and also the USP9X-deficient T cells were hyperproliferative (125). Therefore, E3 ligases and deubiquitinases keep the delicate balance between immunity and tolerance.

## The Therapeutic Targets of UPS and DUBs

Numerous evidence indicates that every component of UPS can be regarded as valuable therapeutic targets in the development of anti-cancer drugs. Several drugs such as bortezomib (a proteasome inhibitor), have been approved by the FDA in cancer, and many other inhibitors are in development (**Table 3**) (138).

**Table 3:** Anti-cancer compounds in clinical trials targeting the ubiquitin-proteasome system and DUBs.

Classification	Compound	Target	Cancer/cancer cell line	Status	References
E1 inhibitor	MLN7243	Ubiquitin-activating enzyme	Acute myeloid leukemia	Phase I/II	(65)
	MLN4924	NEDD8-activating enzyme	Malignant melanoma	Phase I	(52)
	TAK-243	Ubiquitin-like modifier-activating enzyme 1	Acute myeloid leukemia	PreClinical	(42)
	PYR-41	Ubiquitin-activating enzyme	HCT116 cells, H522 cells	PreClinical	(71)
E2 inhibitor	Leucettamol A	Ubc13-Uev1A		Research	(72)
	manadosterols A and B	Ubc13-Uev1A		Research	(91)
	CC0651	Human Cdc34	PC-3 prostate cancer cells, HCT116 cells	PreClinical	(92)
E3 inhibitor	RG7112	MDM2/HDM2	Liposarcoma, acute Leukemia	Clinical	(43)
	RG7388	MDM2	Human osteosarcoma SJSA cells	Clinical	(44)
	SAR405838	MDM2/HDM2	Liposarcoma, gastrointestinal, Melanoma, non-small cell lung cancer	Phase I	(45, 46)
	MK-8242	MDM2/HDM2	Acute myeloid leukemia, Advanced solid tumors	Phase I	(93, 94)
	NVP-CG097	MDM2	SJSA-1 cells	Phase I	(95)
	HDM201	MDM2	Acute myeloid leukemia	Phase I	(96)
	AMG232	MDM2	Solid tumors and lymphomas	Phase I	(97)
	RITA	MDM2	HCT116 cells	Research	(98)
	PRIMA1	MDM2	SW480 tumor, Saos-2 osteosarcoma cells	Research	(99)
	HLI373	HDM2	RPE cells, U2OS cells, MDA-MB-468 breast cancer cell	Research	(100)
	HLI98	MDM2	RPE cells, U2OS cells, LOX-IMVI cells, A549 cells, HT1080 cells	Research	(101)
	MEL23/MEL24	MDM2	U2OS cells, HCT116 cells, RKO cells, HT-1080 cells, H1299 cells, MCF7 cells	Research	(102)
	RO8994	MDM2	SJSA-1 cells, RKO cells, HCT116 cells	Research	(103)
	NSC207895	MDMX	MCF7 cells	Research	(105)
	ATSP-7041	MDM2 & MDMX	SJSA-1 cells, RKO cells, HCT116 cells, MCF7 cell,	Research	(106)
	ALRN-6924	MDM2 & MDMX	Solid tumors and lymphomas	Phase I	(107)
	oridonin	c-Myc	Leukemia and lymphoma cells	Research	(109)
	compound ZL25	SKP2	Prostate cancer cell PC-3 & LNCaP cell, H3255 cells, H1299 cells, Hep3B cells & U2OS cells	Research	(106)
	compound A	SKP2	Hematologic malignancies	Research	(111)
	Erioflorin	Pdcd4	RKO cells, HeLa cells, MCF7 cells	Research	(112)
	GS143	β-TrCP1		Research	(113)
	TAME	Cdh1 and Cdc20	HeLa cells	Research	(114)
	apcin	Cdc20	RPE1 cells	Research	(115)
	Clomipramine	Itch	Breast, prostate and bladder cancer cells	Approved	(118)
Proteasome inhibitor	Bortezomib	Proteasome	Multiple meloma, nonsmall cell lung cancer, pancreatic cancer, mantle cell lymphoma	Approved	(119, 120, 123, 124)
	Carfilzomib	Proteasome	Multiple meloma, Waldenstrom’s Macroglobulinemia	Approved	(7)
	Ixazomib	Proteasome	Multiple meloma	Approved	(8)
	Oprozomib	Proteasome	Multiple meloma, solid tumors, Waldenstrom Macroglobulinemia	Phase Ib/II	(126)
	Delanzomib	Proteasome	Multiple Myeloma, solid tumors, Lymphoma, Non-Hodgkin	Phase I/II	(127)
	Marizomib	Proteasome	Refractory and relapsed multiple myeloma, malignant glioblastoma	Phase III	(126, 128, 129)
DUB inhibitor	WP1130	USP9X	HCT116 cells	Research	(129)
	WP1130	UCH37	Multiple myeloma MM1.S & Mantle cell lymphoma Z138 cells	Research	(130)
	HBX 41,108	USP7	Prostatic adenocarcinoma PC3 cells, Colon carcinoma HCT116 cells	Research	(131)
	P5091	USP7	Multiple myeloma cells	Research	(132)
	b-AP15	USP14 & UCHL5	Multiple myeloma cells	Research	(133)
	Protac-1	MetAP-2		Research	(134)
	ARV-825	BRD4	Multiple myeloma cells	Research	(135)
	ARV-771	pan-BET	Castration-resistant prostate cancer	Research	(136)
	QCA570	BET	Human acute leukemia cells	Research	(137)

### Targeting the E1 Enzyme

The E1 enzyme is responsible for activating ubiquitin molecules in the UPS, and several compounds have been identified to target E1. For example, adenosine sulfamate analogs, such as MLN7243 (126) and MLN4924, function as the ubiquitin-activating enzyme and NEDD8-activating enzyme inhibitors, respectively. They are currently undergoing Phase I/II and Phase I clinical trials (127, 139). Recently, TAK-243 was reported to induce leukemic cell death in preclinical models of acute myeloid leukemia cells through inhibition of the ubiquitin-like modifier-activating enzyme 1 (128). Experimental inhibitors of E1 have also been reported. For example, PYR-41, an irreversible inhibitor of ubiquitin E1, can inhibit the ubiquitylation of TRAF6 and decrease nuclear factor-kappa B activation. PYR-41 can also inhibit the degradation of p53 and activate its transcriptional activity (140). Due to lacking specificity, inhibition of E1 would cause remarkable side effects.

### Targeting the E2 Enzyme

The E2 enzyme binds to E1, and then the activated ubiquitin is transferred to a cysteine of the E2 enzyme from the E1 enzyme. Thus, E2 enzymes mediate the conjugation of ubiquitin to substrates. Nowadays, several E2 inhibitors have been found to interfere with the process. For instance, Leucettamol A and manadosterols A and B, which are isolated from the sea sponges, inhibit the Ubc13-Uev1A interaction and block the E1-E2 complex formation (141, 142). Another example is CC0651, a small molecule inhibitor of the E2 enzyme hCdc34 (130). The E2 enzyme hCdc34 can ubiquitylate SCF (Skp2) substrate p27, and CC0651 decreases tumor cell growth by inhibiting p27 ubiquitylation and degradation.

### Targeting the E3 Enzyme

E3 ligase recognizes substrate proteins and catalyzes the transfer of ubiquitin from E2 to target protein lysine. Therefore, E3 ligase has high substrate specificity which makes targeting E3 ligase become a promising tumor treatment strategy. So far, many studies have identified some compounds that could target specific E3 ligases and disturb UPS.

#### MDM2/p53

Due to the critical roles of p53 in regulating the genome, many efforts have been made to find the antagonists of E3 ligase MDM2/HDM2 to restore the function of p53. To date, a large number of inhibitors have been discovered based on MDM2-p53 interaction. Some of them are undergoing clinical assessment with different stages, such as RG7112 (129), RG7388 (131), SAR405838 (132, 143), MK-8242 (144, 145), NVP-CG097 (133), HDM201 (146), and AMG232 (147). Besides, more MDM2 inhibitors, such as RITA (134), PRIMA1 (135) HLI373 (148), HLI98 (149), MEL23 and MEL24 (150), and RO8994 (136) have been discovered to target MDM2 directly, thereby enhancing p53 activity and exhibiting anti-cancer ability.

MDMX/HDMX (murine/humans double minute X) shares significant homology with MDM2 and is also a negative regulator of p53. Though nutlin-3 has been found to inhibit MDM2-p53 but not MDMX-p53 interaction (151), NSC207895 targets MDMX specifically and acts addictively with nutlin-3a to activate p53 and induce apoptosis (137). Moreover, ATSP-7041 (152) and ALRN-6924 (153) decrease p53-dependent tumor growth as dual inhibitors of MDM2 and MDMX.

#### SCF E3 Ligases

SCF (Skp1/cullin/F-box) E3 ligases are the largest family of E3 ubiquitin ligases. Their substrates play important roles in regulating the cell cycle, DNA replication, and signal transduction. Therefore, the dysregulation of these E3s often leads to cancer (154). Since FBPs are responsible for the specificity of SCFs, many small molecules are designed to target them. For instance, the natural compound oridonin enhances the ubiquitination and degradation of c-Myc mediated by FBW7, inducing apoptosis in leukemia and lymphoma cells (155). Furthermore, compound ZL25 inhibits SKP2 directly, resulting in the p53-independent cellular senescence in cancer cells (156). Another SKP2 inhibitor, compound A, induces p27-dependent cell cycle arrest and cell death by inhibition of SCF-SKP2 complexes formation (157). Erioflorin stabilizes the tumor suppressor Pdcd4 by blocking its interaction with β-TrCP1, suppresses the activity of AP-1 and NF-κB, and decelerates cancer cell proliferation (158). Another inhibitor, GS143, was shown to markedly decrease IκB ubiquitination by targeting β-TrCP1 and suppress the NF-κB signaling pathway (159).

Since Cdc20 is an oncogenic cofactor in the APC/C complex, many efforts have been made to find Cdc20 inhibitors to anti-cancer. TAME (tosyl-L-arginine methyl ester) was reported to bind to the APC complex. It could inhibit its activation by targeting both Cdh1 and Cdc20 and arrest cells in metaphase (160). Moreover, Apcin was found to bind directly to Cdc20, inhibiting the ubiquitylation of D-box-containing substrates, and subsequently inducing tumor cell death (161).

E3 ligase Cbl-b has been identified as a negative regulator of TCR signaling. When Cbl-b is inhibited, the T cell-mediated antitumor activity will be enhanced. Autologous peripheral blood mononuclear cells (PBMCs) from patients were collected and transfected with Cbl-b-siRNA, which were called APN401. The results of the Phase I clinical trial for APN401 revealed that its intravenous infusion in patients with refractory solid tumors was feasible and safe (162). Several small-molecule Cbl-b inhibitors have been discovered to decrease the ubiquitylation of TAM receptors and promote the activation of T cells as well as natural killer cells. They are expected to be utilized in combination with other approved agents in immunotherapy (163).

Itch, a HECT domain-containing E3 ligase, promotes the ubiquitylation of several proteins (e.g. p70, p63, c-Jun, JunB, Notch, and c-FLIP) and shows a potential target for cancer therapy. Rossi et al. identified that antidepressant drug clomipramine and its homologs could inhibit Itch auto-ubiquitylation and p73 ubiquitylation to reduce breast, prostate and bladder cancer cell growth by blocking autophagy (164).

### Targeting Proteasome Activity

Among all the UPS components, the proteasome has been successfully used as a target for cancer treatment. The proteasome is a large multi-protein complex containing multicatalytic proteases (e.g., chymotrypsin- and caspase-like enzyme) and is responsible for the degradation or processing of intracellular proteins. As such, it regulates the levels of some important mediators for cell-cycle progression and apoptosis in normal and malignant cells, such as cyclins, caspases, BCL2 and nuclear factor of κB (165). Bortezomib is the first proteasome inhibitor approved for recurrent refractory multiple meloma (MM) in 2003 (166, 167). It reversibly inhibits the activities of chymotrypsin- and caspase-like enzymes, leads to the apoptosis of MM cells, and suppresses the activation of NF-κB, production of cytokines (e.g., IL-6, IGF-1, and VEGF) in the tumor microenvironment, and adherence of myeloma cells to bone marrow stromal cells (165, 168). Later, it was extended to patients with non-small cell lung cancer, pancreatic cancer, and mantle cell lymphoma (169, 170). Although bortezomib has antitumor activity, it can cause side effects such as neuropathy and autophagy in some cases (171, 172). Besides, bortezomib resistance often occurred in about one year (173, 174). Carfilzomib, a second-in-class proteasome inhibitor drug, was approved in 2012 for MM by the US FDA (11). It irreversibly inhibits the chymotrypsin-like activities and shows improved safety in maintaining its cytotoxic potential in the bortezomib resistant cell lines (12). Carfilzomib treatment also causes adverse effects such as cardiovascular complications, hypertension, and heart failure, but they are reversible and manageable with careful monitoring. Both bortezomib and carfilzomib are not suitable for oral administration. Ixazomib is the first oral bioavailable proteasome inhibitor and was approved by the FDA in 2015. It reversibly inhibits the chymotrypsin-like activities and shows improved safety profiles over bortezomib, but its therapeutic advantages still need further investigation by randomized clinical trials (12).

The clinical successes of existing proteasome inhibitors encourage great efforts to discover more proteasome inhibitors with improved efficacy and safety. Thus, a lot of proteasome inhibitors have been identified including oprozomib, delanzomib and marizomib. Oprozomib is an orally available inhibitor with a homologous structure to carfilzomib. It is currently being studied in several clinical trials including a multicenter phase Ib/II trial for MM patients. Oprozomib can effectively decrease the viability of MM cells both *in vitro* and *in vivo* (175). Delanzomib, a reversible oral bioavailability of bortezomib analog, overcomes bortezomib’s resistance to peripheral neuropathy. But it causes severe skin toxicity to many patients (176). Marizomib, a novel proteasome inhibitor with a better therapeutic ratio, overcomes bortezomib resistance and exhibits broader anti-cancer activities (177). Moreover, marizomib has synergistic effects on refractory and recurrent MM patients with BTZ, linedoxamine, bormadoxamine and low dose dexamethasone (175, 178). In addition, marizomib can penetrate the blood-brain barrier and induces apoptosis in glioma cells with low toxicity on normal cells (179). Marizomib is currently being assessed in a phase III trial for the treatment of malignant glioblastoma in combination with temozolomide and radiotherapy.

### Targeting DUBs Activity

Ubiquitination is a dynamic and reversible process and DUBs catalyze the removal of ubiquitin or polyubiquitin chains from the target protein. DUBs are actively involved in regulating tumorigenesis. Thus, DUB inhibitors are regarded as potential anti-cancer agents (180) To date, a number of DUB inhibitors have been identified to inhibit tumorigenesis (4, 10, 181).

WP1130, an inhibitor of DUBs, can suppress the activities of USP9X, USP5, USP14 and UCH37, deregulate anti-apoptotic protein MCL-1 and upregulate pro-apoptotic protein p53. It exhibits high anti-tumor activity (182). For example, the transcription factor E-twenty-six related gene (ERG) is overexpressed and promotes prostate carcinogenesis. Inhibition of USP9X by WP1130 leads to ERG degradation and inhibits tumor growth (183).

Recently, HBX 41,108, a small-molecule inhibitor of USP7, was reported to inhibit USP7-mediated p53 deubiquitination, stabilizing p53 and inducing p53-dependent apoptosis in cancer cells (184). Besides, P5091, a selective USP7 inhibitor, was found to induce apoptosis and overcome bortezomib resistance in MM cells. What’s more, it can inhibit tumor growth and exhibit synergistic anti-MM activity in combination with lenalidomide, HDAC inhibitor SAHA, or dexamethasone (185). A class of dual small molecule inhibitors of USP7 and USP47 has been identified to promote p53 activity and apoptosis in MM and B-cell leukemia cells *in vitro* and xenograft models (186).

Moreover, USP14 can inhibit the degradation of ubiquitin-protein conjugates *in vitro* and *in vivo* (187). The inhibitors of USP14 have been found to stimulate the proteasomal degradation of oxidized proteins, causing resistance to oxidative stress (188). Consistently, b-AP15 was shown to inhibit cell growth and overcome bortezomib resistance in MM cells by selectively blocking the deubiquitylating activity of USP14 and UCHL5 (189). These studies indicate that inhibiting specific oncogenic DUBs may be an effective anti-cancer approach.

## PROTACs Technology

**Figure 5:**
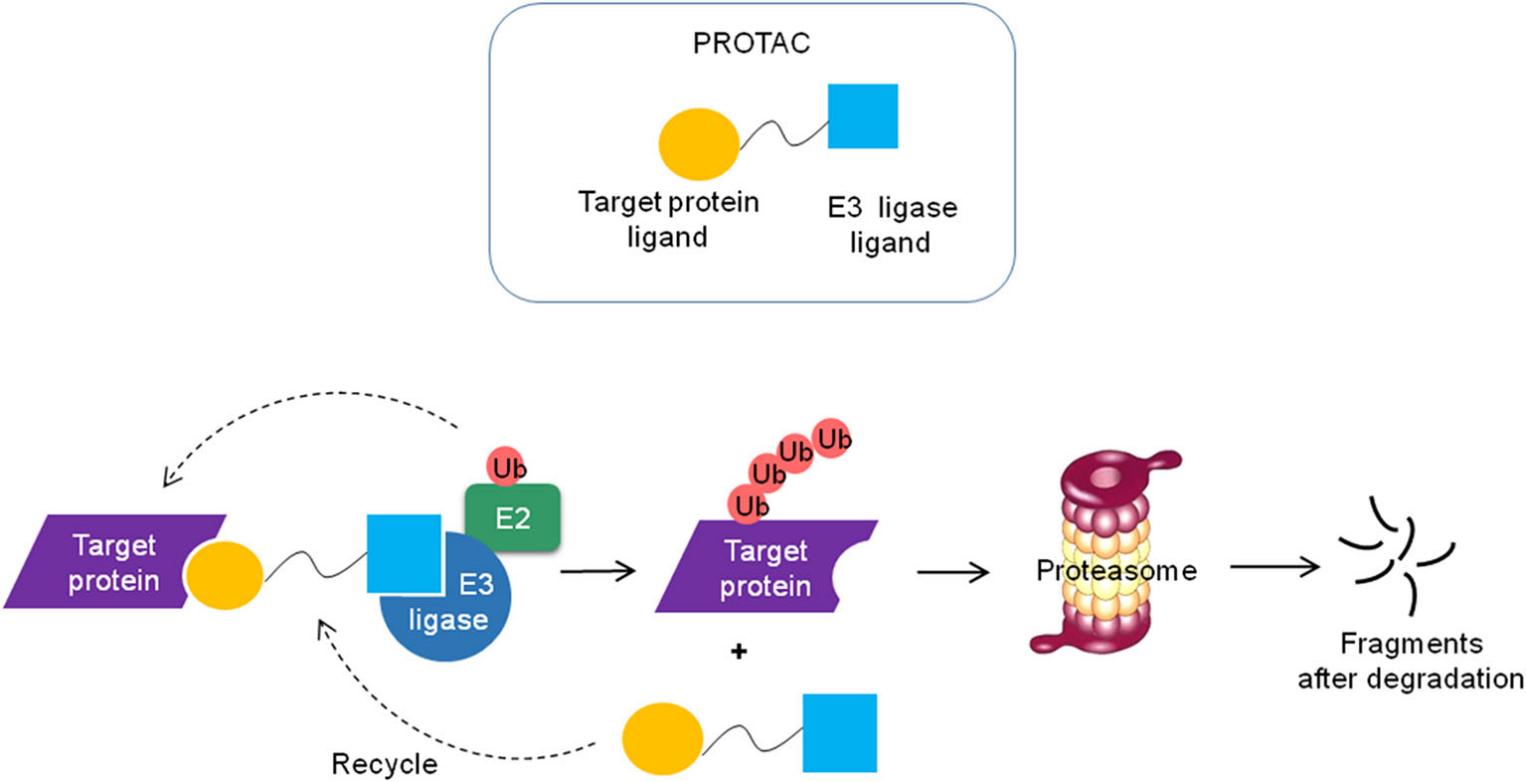
Schematic diagram of the PROTAC technology. The PROTC is a chimeric molecule that consists of two ligands, one is to interact with E3 ligase and the other is to bind the target protein. The target protein is polyubiquitinated and degraded by the proteasome and the PROTC molecule can be recycled.

Recently, emerging technologies based on PROTACs attract increasing attention in the pharmaceutical industry (190). PROTACs are heterobifunctional molecules that simultaneously bind a target protein and an E3 ubiquitin ligase, enabling ubiquitination and degradation of the target by the UPS in the cell (**Figure 5**) (13). PROTACs link the target protein to an E3 ubiquitin ligase by a designed hybrid molecule, providing a path for ubiquitinating undruggable proteins such as transcription factors, scaffolding proteins and nonenzymatic proteins. The first PROTACs were reported in 2001 by the Crews group and Ray Deshaies (191). They artificially synthesized a chimeric compound named Protac-1. Protac-1 has two domains: one domain contains the IκBa phosphopeptide that could recruit the F-box protein β-TrCP, and the other domain contains ovalicin which could bind to the target protein methionine aminopeptidase-2 (MetAP-2). As a result, MetAP-2 was ubiquitinated and degraded in a Protac-1-induced proteolysis manner.

Due to the excellent permeability and low working concentrations, small molecule-based PROTACs, which utilize small molecules to recruit E3 ubiquitin ligases, have more potential to be developed into drugs than peptide-based PROTACs (13). The PROTAC technology broadens the range of target proteins degraded by the UPS.

Recently, some transcriptional regulators (such as BRD4, TRIM24 and Smad3) have been reported to be targeted by PROTAC technologies (13). BRD4, a bromodomain and extraterminal domain (BET) family member, usually resides upstream of important oncogenes such as c-Myc, BCL-xL and BCL-6, and regulates their expressions. Therefore, BRD4 has become a promising therapeutic target in multiple cancer types. Preclinical studies of BRD4 inhibitors, JQ1 and OTX015, demonstrate their value in suppressing c-Myc expression and BL cell proliferation. However, owing to the reversible binding of inhibitors, the suppression is incomplete and requires high drug concentrations. Crews groups developed a bifunctional molecule, ARV-825, connecting the BRD4 inhibitor OTX015 to an E3 ligase cereblon binding moiety (pomalidomide) using PROTAC technology. As a result, ARV-825 actively recruits BRD4 to cereblon, leading to BRD4 efficient degradation *via* the proteasome in Burkitt’s Lymphoma cells. Moreover, ARV-825 treatment produces a more pronounced effect on the inhibition of c-Myc than that of the BRD4 inhibitors in five MM cell lines [SKO-007(J3), U266, RPMI-8226, ARP-1, JJN3] and an MM patient-derived CD138^+^ MM cells (192). In addition, Zengerle et al. designed another PROTAC, connecting JQ1 for BET family proteins and a ligand for VHL. Interestingly, the PROTAC not only triggered the degradation of BET family proteins particularly BRD4, but also regulated the transcription of BRD4 downstream genes such as Myc, p21 and AREG (193). In this way, it can also dampen the pro-inflammatory response in microglia, because BET proteins control the transcription of NF-κB-depended genes (194). These findings demonstrate that BRD4 PROTACs is a promising novel strategy to efficiently target BRD4 (195).

Raina and his colleagues reported that ARV-771 (another pan-BET inhibitor)-based PROTAC, dramatically suppressed androgen receptor (AR) protein level and AR signaling. It could lead to tumor regression in castration-resistant prostate cancer (CRPC) mouse xenograft model with more efficiency than BET inhibitors. This study provides evidence that small molecule-based PROTAC functions in a solid-tumor malignancy of CRPC (196). The results of BET-PROTACs ARV-825 and ARV-771 in the treatment of MCL cells demonstrate that they induce more apoptosis than BET inhibitors. Also, the results show that they can overcome the resistance of ibrutinib and exert a synergistic effect on apoptosis induction in the combination of other drugs such as ibrutinib, venetoclax (a BCL2-antagonist) and palbociclib (a CDK4/6 inhibitor) (197).

Recently, more BET-PROTACs have been designed. For instance, Qin et al. synthesized a BET-PROTAC called QCA570, utilizing a new class of BET inhibitors Oxazepines to recruit BET proteins. It could inhibit human acute leukemia cell proliferation at low picomolar concentrations, and abolish tumor growth in leukemia xenograft models in mice (198). Zhang and his colleagues demonstrated that BET-specific PROTACs were active against preclinical models of MM (199). Interestingly, the activity of BRD4-specific PROTACs can be improved over 100-fold through modification of hydroxylation of proline (200). In addition to the BET family, a functional PROTAC against TRIM24, another bromodomain-containing transcriptional regulator, has been designed and provides a path to find new undruggable targets (201). Wang et al. designed new PROTACs to prevent renal fibrosis by targeting SMAD3. They used hypoxia-inducible factor-1α to recruit VHL and screened compounds to bind SMAD3 from the Enamine library using the GLIDE molecular docking program. SMAD3 was degraded by PROTAC mediated ubiquitination (202). Thus, transcription factors can be targeted *via* PROTAC technology.

In addition, the undruggable transcription factors also can be degraded *via* alteration of the activity of an E3 ubiquitin ligase. For instance, Thalidomide and its derivatives Lenalidomide and Pomalidomide are effective drugs for the treatment of multiple myeloma and other B cell lymphomas. Thalidomide analogs bind Cereblon (CRBN), the substrate receptor of the CUL4-RBX1-DDB1-CRBN (CRL4CRBN) E3 ubiquitin ligase and alter its substrate selectivity to recruit, ubiquitinate and degrade unrelated transcription factors, such as Ikaros (IKZF1), Aiolos (IKZF3) and Casein kinase 1 alpha (CK1α) (203, 204). These findings provide a novel way to selective degrade specific targets through modulating the activity of an E3 ubiquitin ligase.

## Conclusions

Ubiquitination of nonhistone proteins plays an important role in many cellular processes, including cell cycle, cell proliferation, DNA repair, apoptosis, inflammation, immune response, etc. Dysregulation of nonhistone lysine ubiquitylation is closely associated with the development of various human cancers. Therefore, UPS has been evolved as promising therapeutic targets for novel anti-cancer drugs. Nowadays, many proteasome inhibitors and E3 ligase modulators have been approved for anticancer treatment, whereas small-molecule inhibitor therapeutic strategies usually need high drug exposures and potentially increase the risk of off-target adverse effects. Fortunately, PROTAC technologies provide a path to target many undruggable proteins with UPS such as transcription factors.

To date, it remains an obstacle for the discovery of small molecule moiety to different targets. Another obstacle is specificity, how to get tissue-specific or disease-specific induced protein degradation? How to realize conditional triggered induced protein degradation? A deeper understanding of the tissue expression of E3 ligase and tumor microenvironment may provide a larger therapeutic window for appropriate PROTAC.

## Author Contributions

XZ wrote and drafted this article. TM and LF revised the manuscript critically. SC and DL drew the figures and prepared the table. QP and PW contributed to the drafting of the article and are responsible for the integrity of the work as a whole. All authors contributed to the article and approved the submitted version.

## Funding

This work was supported by the Natural Science Foundation of Shandong Province (No. ZR2016CM46).

## Conflict of Interest

The authors declare that the research was conducted in the absence of any commercial or financial relationships that could be construed as a potential conflict of interest.
